# Quality control for community-based sea-ice model development

**DOI:** 10.1098/rsta.2017.0344

**Published:** 2018-08-20

**Authors:** Andrew F. Roberts, Elizabeth C. Hunke, Richard Allard, David A. Bailey, Anthony P. Craig, Jean-François Lemieux, Matthew D. Turner

**Affiliations:** 1 Naval Postgraduate School, Monterey, CA, USA; 2 Los Alamos National Laboratory, Los Alamos, NM, USA; 3 Naval Research Laboratory, Stennis Space Center, MS, USA; 4 National Center for Atmospheric Research, Boulder, CO, USA; 5 Cherokee Nation Technologies in support of NOAA Earth System Research Laboratory, Washington, DC, USA; 6 Recherche en Prévision Numérique Environnementale, Environnement et Changement Climatique Canada Dorval, QC, Canada; 7 DoD HPCMP PETTT, Engility Corp., Stennis Space Center, MS, USA

**Keywords:** CICE, Icepack, earth system modelling, sea-ice forecasting

## Abstract

A new collaborative organization for sea-ice model development, the CICE Consortium, has devised quality control procedures to maintain the integrity of its numerical codes' physical representations, enabling broad participation from the scientific community in the Consortium's open software development environment. Using output from five coupled and uncoupled configurations of the Los Alamos Sea Ice Model, CICE, we formulate quality control methods that exploit common statistical properties of sea-ice thickness, and test for significant changes in model results in a computationally efficient manner. New additions and changes to CICE are graded into four categories, ranging from bit-for-bit amendments to significant, answer-changing upgrades. These modifications are assessed using criteria that account for the high level of autocorrelation in sea-ice time series, along with a quadratic skill metric that searches for hemispheric changes in model answers across an array of different CICE configurations. These metrics also provide objective guidance for assessing new physical representations and code functionality.

This article is part of the theme issue ‘Modelling of sea-ice phenomena’.

## 1. Introduction

Sea ice is a critical component of the Earth system, governing the high-latitude surface radiation balance and atmosphere–ocean exchanges of heat, moisture and momentum. It forms a formidable navigational hazard, occurs in some of the most biologically productive seas on Earth, and covers 7–10% of the ocean in the current epoch. For these reasons, there is a strong need to accurately simulate its thickness, concentration and velocity on daily to centennial timescales for global weather and climate prediction, as well as maritime operations. Since the late 1990s, the Los Alamos Sea Ice Model (CICE) has provided a platform for international collaboration in the development of new sea-ice model physics and numerics for massively parallel supercomputers. CICE is used in more than 20 countries to research sea-ice processes and their interactions with the climate system, in 12 coupled models used for the Intergovernmental Panel on Climate Change Fifth Assessment Report [1], and in operational settings by the US Navy [2], Environment and Climate Change Canada (ECCC) [3] and other forecasting centres. Two main reasons for CICE's widespread use is that it is computationally efficient for simulating the growth, melt, and movement of sea ice, and contributions to the model are transparent and subject to peer review by virtue of its extensive user base and documentation.

During the past two decades, members of the sea-ice modelling community have contributed significant capabilities to CICE, including physical parameterizations, infrastructure elements such as different types of grids, and parallel computational performance improvements. Since the release of CICE v. 5 in 2015 [4], the model has undergone substantial architectural enhancements in the form of a new ‘Icepack’ submodule. Icepack contains the biogeochemistry and model physics that are necessary to simulate frozen ocean in individual model grid cells, such as ice ridging, thermodynamics and thermohaline hydrology [5,6]. Icepack interfaces seamlessly with the CICE dynamical core, which includes momentum, advection and the elastic-viscous-plastic (EVP) [7–9] and elastic-anisotropic-plastic (EAP) [10] rheologies. With Icepack, CICE column physics can now be used separately in earth system models along with a different dynamical sea-ice core. Icepack also can be used to synthesize Lagrangian field measurements.

In 2016, the primary developers and users of CICE founded the CICE Consortium, formalizing and enhancing long-standing collaborations to foster sea-ice model advances for research and operational applications. The Consortium developed a governance structure within an open software development environment, along with mechanisms to ensure that its codes remain portable, flexible, extensible, robust and well documented. As part of this structure, we have established an objective method to arbitrate changes to the CICE code, the subject of this paper.

A central tenet of our stewardship of CICE is the modeller's equivalent of the Hippocratic Oath: additions and changes to CICE must not alter the answers of existing model configurations unless correcting scientifically proven errors or bugs, or updating the physics, biogeochemistry, numerics or parameter space of the model based on new research. This development criterion is more onerous than it may seem, because CICE facilitates configurations so different from one another that they may barely be considered the same sea-ice model. The code is currently configurable with one of three rheologies [8–10], three vertical thermodynamic models [11–13], three melt pond representations [14–16] and two radiation schemes [17,18], among a broader sweep of run-time options described in the model documentation [5,6]. Consequently, new additions to CICE will likely alter existing code, which in turn may relinquish bit-for-bit (BFB) reproducibility of enduring configurations. If BFB reproducibility cannot be achieved when new model additions are switched off, we must then determine whether the non-BFB changes *significantly* alter existing model configurations, including the dozens of possible configurations highlighted above.

Here, we describe an efficient and automated acceptance testing method for controlling the quality of new contributions to CICE. We seek a method that quickly scrutinizes non-BFB changes in efficient, stand-alone CICE-Consortium code as a first verification against inadvertent bugs or numerical inaccuracies. The method must be independent of computer platforms, compilers and their optimizations. A need for this tool frequently arises in model development, for example when a new physics option requires the re-ordering of operations in an existing model equation, or it introduces a quotient to an existing model equation that is analytically but not numerically identical to its previous implementation. Our method exploits statistical properties of sea-ice thickness evolution common across a range of sea-ice models, both stand-alone and coupled, which we describe in §2. The quality control measures are described in §3. Section 4 presents examples and discussion of the method using CICE6, and compares quality-control results from ostensibly identical but non-BFB CICE6 codes against climate- and physics-altered examples. Section 5 contains a brief conclusion.

## 2. Model data used in this study

Understanding whether or not non-BFB changes in CICE code may also alter the climate of the model can be non-trivial. By ‘climate changing’, we mean significant changes in sea-ice thickness, *h*, over a substantial fraction of the ice pack within a defined number of annual cycles. *h* integrates changes in sea-ice growth, melt, drift and deformation, and therefore the time series *h*_*i*_ of ice thickness, weighted by ice concentration, documents evolution of simulated ice mass and underpins our quality control (QC) procedure (*i* is a time index). Currently, 5 years or less is the lifespan of much of the perennial ice in the Arctic and surrounding the Antarctic, and we define that period as the time range over which non-BFB climate-changing signals must emerge. Coincidentally, 5-year CICE integrations are short enough to enable dozens of model configurations to be routinely interrogated overnight; longer integrations are more taxing of the available computing resources, and a shorter test window risks missing emergent signals in *h*_*i*_. Therefore, we seek to exploit common statistical features observed in semi-decadal sea-ice model integrations to design a technique sensitive enough to flag answer changes across a diverse range of CICE implementations and ice-covered seas.

**Figure 1.**
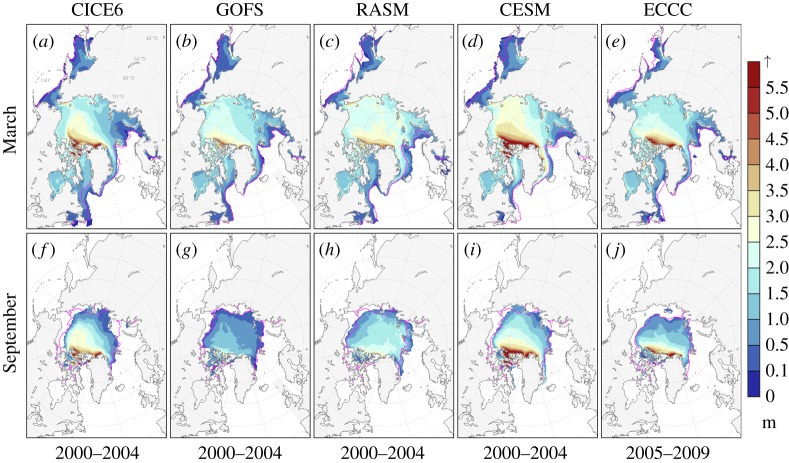
(*a*–*e*) March and (*f*–*j*) September Arctic mean 0000 UTC sea-ice thickness from five models summarized in table 1 for years 2000–2004 except for ECCC, which are for 2005–2009. CESM large ensemble averages are constructed from the first five ensemble members. Thicknesses are plotted only for model sea-ice concentrations greater than 15%. Magenta contours indicate observed mean March and September sea-ice extent calculated from the NOAA/NSIDC Climate Data Record [25].

The CICE Consortium models used to identify universal statistical ice-mass properties are summarized in table 1, and their mean Arctic thickness results are illustrated in figure 1 as evidence of suitability for this study. These results were obtained from *h*_*i*_ time series of daily 0000 UTC mean or instantaneous model output. Our core 5-year study period is 2000 through 2004, using CICE6, GOFS, RASM and CESM. We also use a 2005–2009 ECCC integration as evidence that the uniform statistical signals among the other models are not biased by the chosen study period. The diverse set of model configurations used here helps ensure that the statistical properties we observe are not merely due to the type of model used, be it uncoupled, forced ice–ocean, assimilated, or fully coupled. We briefly elaborate on each model's configuration to highlight that diversity:

**Table 1.** Summary of sea ice models used in this study.

model^a^	lead^b^	configuration^c^	domain	CICE^d^	thermodynamics [12,19]	radiation [17,18]	melt ponds [14,15]	dynamics^e^
CICE6 [5,6]	LANL	ice	global	6.0	Mushy Layer	Delta-Eddington	Level Ice	EVP
GOFS [2]	NRL	ocn-ice-assim	global	4.0	Bitz–Lipscomb	CCSM3	—	EVP
ECCC [3,20]	ECCC	ocn-ice	regional	4.0	Bitz–Lipscomb	CCSM3	—	EVP/landfast Ice
RASM [21,22]	NPS	ocn-ice-atm-lnd	regional	5.1	Mushy Layer	Delta-Eddington	Level Ice	EVP and EAP
CESM [23]	NCAR	ocn-ice-atm-lnd	global	4.1	Bitz–Lipscomb	Delta-Eddington	CESM	EVP

^a^CICE6, CICE Consortium dynamic core with Icepack; GOFS, US Navy Global Ocean Forecast System v. 3.1; ECCC, Environment and Climate Change Canada model; RASM, Regional Arctic System Model v. 1.1; CESM, Community Earth System Model Large Ensemble.^b^LANL, Los Alamos National Laboratory; NRL, Naval Research Laboratory; ECCC, Environment and Climate Change Canada; NPS, Naval Postgraduate School; NCAR, National Center for Atmospheric Research.^c^ice - standalone sea ice model; ocn-ice - coupled ocean and ice model forced with atmospheric reanalyses; ocn-ice-assim - assimilated and coupled ocean and ice model forced with atmospheric reanalyses; ocn-ice-atm-lnd - fully coupled ocean, sea ice, atmosphere and terrestrial models, forced laterally with observation-based datasets if regional, or with transient greenhouse gas concentrations if global.^d^CICE code v. 4 [24], 5 [4] or 6 [5,6].^e^EVP, elastic-viscous-plastic [7,8]; EAP, elastic-anisotropic-plastic [10].

*CICE6*: Los Alamos National Laboratory's stand-alone configuration of CICE v. 6.0.0.alpha [5,6] was run on an efficient ‘*gx1*’ (1°) global, displaced-pole test grid using 1 h time steps. Sea surface temperature was computed with a slab ocean mixed layer forced by derived atmosphere and ice fluxes along with monthly climatological ocean model output as described in [26]. This configuration was spun up from 1990 to 1999, starting from CICE's default restart data, and *gx1* analysis runs from 2000 onwards were initialized using that integration, including decadal simulations introduced in §4.

*GOFS*: The Global Ocean Forecast System (GOFS 3.1) [2] consists of the HYbrid Coordinate Ocean Model (HYCOM) coupled to CICE v. 4.0. Both models share a common tripole horizontal grid with approximately 3.5 km resolution at the North Pole. The Navy Coupled Ocean Data Assimilation used in 2000–2004 reconstruction employs a 3D multivariate ocean data assimilation scheme for satellite-derived sea surface height and temperature, sea-ice concentration, and *in situ* subsurface ocean observations. This reanalysis was initialized from a 9-year global HYCOM/CICE simulation run with climatological forcing and was forced with NCEP CFSR/CFSRV2 atmospheric forcing [27,28] for a 17-year period beginning 1 October 1998.

*ECCC*: The Canadian pan-Arctic ice–ocean model output comes from a 10-year simulation (October 2001–December 2010), over which the period up to October 2004 was used for spin up. The 0.25° regional grid, a subset of the global ORCA mesh [29], covers the Arctic, the North Atlantic and the North Pacific. ECCC uses CICE v. 4.0 [24] with some important modifications that include a grounding scheme and a modified EVP rheology [20], with ice strength based on [30] using 10 ice thickness categories. The ocean model is NEMO v. 3.6, applied in a variable volume and nonlinear free-surface configuration with 13 tidal constituents. The ice–ocean simulations were forced by 33-km resolution atmospheric re-forecasts [31], and ocean boundary conditions are from the GLORYS2V4 reanalysis [32]. The simulations were initialized with average September–October 2001 ice concentration from the National Snow and Ice Data Center [33] and average October–November 2003 sea-ice thickness field derived from ICESat data [34]. The ICESat thickness (mean thickness in a grid cell) was distributed among 10 model thickness categories using a parabolic function. The ocean was started at rest with unperturbed surface height and initial temperature and salinity averaged from September–October WOA13-95A4 fields [35].

*RASM*: v. 1 of the Regional Arctic System Model employs CICE v. 5.1 with a near-identical configuration as in the stand-alone CICE6 model. The baseline configuration uses EVP, and we also include a previously published simulation using EAP [21,22]. RASM's sea-ice component includes inertial-resolving (20 min) coupling with atmospheric, ocean and land components [36], the Weather Research and Forecasting Model, the Parallel Ocean Program (POP) and the Variable Infiltration Capacity run-off model, respectively. The regional configuration, coupling infrastructure and lateral boundary conditions follow [21,22]. The simulations were initialized from a spun-up ocean in 1979, from which we have extracted data for the core 2000–2004 study period, as well as 1996–2000 time series introduced in §3b.

*CESM*: Community Earth System Model data comes from the Large Ensemble Community Project [23], using the fully coupled, global configuration of CESM v. 1 with all components at the nominal 1° global resolution. The Community Atmosphere Model v. 5 and the Community Land Model v. 4 were run on a finite volume grid with 30 vertical levels in the atmosphere. The CICE (v. 4.1) and POP ocean models were run on the *gx1* grid. The spinup procedure involved a multi-century control run with near-zero top-of-the-atmosphere energy balance and 1850 repeated annual cycle of greenhouse gases, solar, and other forcing. One twentieth century ensemble member was branched from year 401 of the control run, using 1850-to-the-present estimates of greenhouse gases, solar, volcanic and other forcing. Additional runs were branched from year 1920 of this simulation to complete the twentieth century ensemble. Each was initialized with a round-off perturbation in the initial surface air temperature, otherwise identical to the others. The first five ensemble members are sufficient to establish that our statistical inferences are robust among the multi-model ensemble in table 1. While much of our analysis is focused on the Arctic, we use CESM to demonstrate that the statistical properties of sea ice used in CICE quality control are equally applicable to Southern Ocean simulations.

## 3. Method of quality control

Changes, additions and updates to CICE fall into four categories: (I) BFB with no further assessment required; (II) non-BFB but unlikely to be climate changing; (III) non-BFB and climate changing; and (IV) a new model configuration option requiring separate scientific assessment. This section describes the automated methods used to flag the first three categories. The control measures provide diagnostic tools to help evaluate code flagged at Category II or above. Category IV contributions are subject to scientific review by the Consortium, but may be assessed using the same statistical tools used to differentiate modifications falling into Categories I, II and III.

### (a) Bit-for-bit reproducibility

Simple BFB benchmarking is commonly enforced in Earth System Modelling projects to prevent avoidable errors entering a code base, by comparing approximately 10-day integrations of modified code against benchmarked histories. BFB tests pass when there is an exact replication of previous results at the level of computational accuracy, placing the suggested code modifications into Category I. If the results are not BFB, testing progresses to the Two-Stage Paired Thickness Test (§3b) after first being reviewed for obvious flaws or avoidable numeric inaccuracies.

### (b) Two-stage paired thickness test

This test quantifies the total fraction of a simulation's sea-ice domain in which the mean thickness is significantly different from that of a defined CICE baseline. First, it tests the difference between the time average of two concentration-weighted thickness time series, *h*_*i*_, in each model grid cell for a baseline ‘*a*’ simulation against a modified ‘*b*’ integration. Then, we determine the total fraction of the sea ice domain with a statistically significant difference, and use it as a measure of whether or not the climate of the model has been perturbed beyond a defined threshold.

A standard *t*-test could be used to determine whether or not two means are statistically different for their paired *h*_*i*_ series *h*_*ai*_ and *h*_*bi*_ in each grid cell for simulations *a* and *b*, respectively, if the samples at each time level *i* are independent of one-another:
3.1
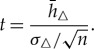
Here, the difference between two means, 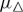, is estimated as 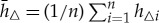 for *n* paired daily samples where the subscript 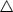 indicates a paired difference obtained from 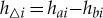 with variance 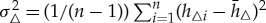. A standard *t*-test would confirm the null hypothesis, 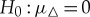, if |*t*| < *t*_crit_(1 − *α*/2, *N*) for degrees of freedom *N* = *n* − 1 at the *α* significance level obtained from a regular *t*_crit_ tabulation; two-sided 80 and 95% confidence intervals have respective values *α* = 0.2 and 0.05. The problem with using equation (3.1) is that sea-ice thickness time series possess such a high degree of autocorrelation that the standard *t*_crit_ values can give an inaccurate indications of whether not the null hypothesis, 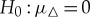, or the alternate hypothesis, 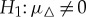, is true. If *H*_0_ is true, we confirm that two simulations' climates are ostensibly identical in a model grid cell, or conversely if *H*_1_ is true, we confirm they are not.

**Figure 2.**
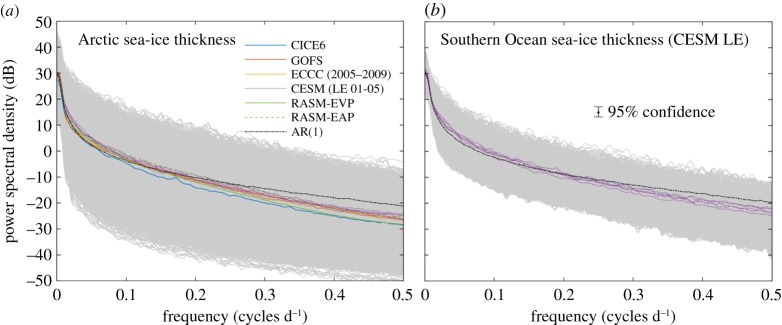
Power spectral density of perennial concentration-weighted sea-ice thickness *h*_*i*_ (*a*) for the Arctic for all models and (*b*) for the Southern Hemisphere from the CESM Large Ensemble. Spectra of individual model grid cells are displayed in grey, and the mean of each simulation's spectra appear in colour, including individual traces for CESM ensemble members 01 to 05. All spectra represent the period 2000–2004 (except for ECCC, 2005–2009). The GOFS spectral mean has been obtained by sampling one model cell per each 10 × 10 grid point mat due to the resolution of that model. RASM spectra are shown for two rheological configurations: EVP as in figure 1, and EAP, corresponding to previously published results [21,22]. Converged Monte Carlo AR(1) spectral estimates appear in black, and the confidence interval displayed in (*b*) also applies to (*a*). The limited spread of spectra for the Southern Ocean relative to the Arctic is due to the comparatively small area of perennial ice that occurs in the Southern Hemisphere.

The extent of autocorrelation in sea-ice thickness is evident in the figure 2 spectra of 5-year *h*_*i*_ time series for every model grid point with perennial sea ice in CICE6, ECCC, CESM and RASM, and every 100th grid point from GOFS owing to that model's resolution. The spectra were calculated using the autocovariance method [37], and the coloured traces provide the mean for each model. We have removed seasonal ice from this analysis to avoid ambiguity introduced by time series with heterogeneous zero-thickness segments. However, we have independently verified that each model's seasonal ice thickness spectra are similarly characterized by the red-noise properties exhibited in figure 2. That characteristic, combined with the uniformity of the spectral means (figure 2), occurs irrespective of model configuration, coupling or forcing, ensemble member, hemisphere, physics options or 5-year window. It demonstrates that a first-order autoregressive (AR(1)) process is a robust approximation of *h*_*i*_ evolution. The black traces in figure 2 provide an AR(1) fit to the ensemble mean of 100 spectra from time series of length *n* = 1825 given by
3.2
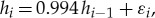
for the white noise process *ε*_*i*_. Respective AR(1) spectral means from equation (3.2) are plotted in figure 2*a*,*b* with the same direct current (DC) offset as the multi-model ensemble spectral average for the Northern and Southern Hemispheres. Equation (3.2) conveys the high level of autocorrelation inherent in all of the spectra seen in figure 2, as demonstrated by their sharp drop-off from the zero-frequency peak, which is a classic red-noise signal.

For such strong statistical dependence between samples in *h*_*ai*_ and *h*_*bi*_, it is common to adjust the definition of *t* in equation (3.1) but still use regular *t*_crit_ look-up tables. As AR(1) is a reasonable statistical model of *h*_*i*_, we may use a *t*-statistic with an effective sample size *n*_eff_ = *n*(1 − *r*_1_)/(1 + *r*_1_) and degrees of freedom *N* = *n*_eff_ − 1 given the lag-1 autocorrelation *r*_1_ [38]:
3.3
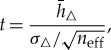
constrained by *n*_eff_∈[2, *n*]. However, there still remains a flaw in this method when *n*_eff_ < 30 [39]; the *t*-test in equation (3.3) becomes conservative for highly autocorrelated series, meaning that *H*_0_ may be erroneously confirmed [39]. In CICE6 simulations presented in this paper, as much as 84, 33 and 14% of the sea ice zone met the *n*_eff_ < 30 criteria for 1-, 5- and 10-year simulations, respectively, and between 65 and 82% of ice-covered areas possessed *r*_1_≥0.9. To counter such problems, Zwiers & von Storch (ZVS) [39] devised a way of checking whether or not the null hypothesis is erroneously confirmed when *n*_eff_ < 30 in equation (3.3).

**Figure 3.**
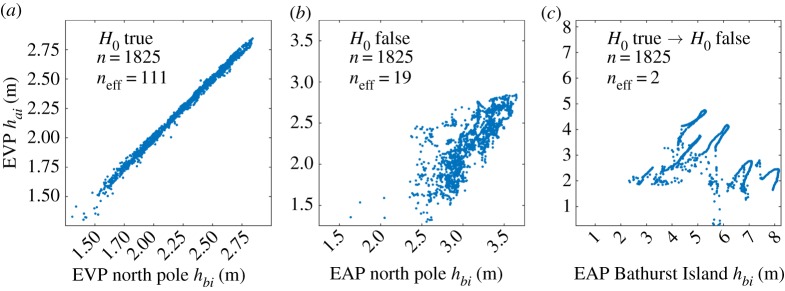
Demonstration of the Two-Stage Paired Thickness Test (2SPT) for daily concentration-weighted ice thickness series from the Regional Arctic System Model (RASM) extending for 5 years from 1996 to 2000 (*n* = 1825): (*a*) The Stage-1 *t*-test in equation (3.3) confirms the null hypothesis, 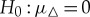, for *h*_*ai*_ and *h*_*bi*_ taken from adjacent grid cells at the North Pole for the EVP simulation. (*b*) The *t*-test in equation (3.3) confirms the alternate hypothesis, 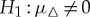, for respective *h*_*ai*_ and *h*_*bi*_ series co-located at the North Pole for EVP and EAP simulations, so that the test stops at Stage-1 even though *n*_eff_ < 30. (*c*) The test proceeds to Stage 2 because *n*_eff_ < 30 for co-located perennial ice time series north of Bathurst Island and the *t*-test in equation (3.3) confirms *H*_0_. The Stage-2 test using equation (3.1) with the look-up table in table 2 subsequently corrects the outcome to confirm the alternate hypothesis, *H*_1_. Time series locations used in this figure are tagged in figure 4*h* in magenta. (Online version in colour.)

To demonstrate the ZVS method as we apply it to CICE, we use examples from 5-year paired *h*_*i*_ series at specific locations on the RASM grid. In the first case in figure 3*a*, we have taken *h*_*i*_ from adjacent grid points near the North Pole in the RASM EVP simulation and plotted them against one another as *h*_*ai*_ and *h*_*bi*_. In this case *n*_eff_ = 111 and our test of the difference of their means using equation (3.3) and a standard *t*_crit_ look-up table confirms the null hypothesis *H*_0_ at the 80% confidence interval. In the second example (figure 3*b*), we compare co-located North Pole time series of the RASM EVP and EAP simulations, which clearly possess different time-averaged ice thickness. In this case, the test using equation (3.3) is flagged as potentially erroneous, because *n*_eff_ = 19, but the *t*-test using equation (3.3) confirms *H*_1_, and the standard test, adjusted for autocorrelation, has worked. In the third example (figure 3*c*), and for the same pair of EVP/EAP simulations, time series north of Bathurst Island are highly autocorrelated, resulting in *n*_eff_ = 2. In this case, *H*_0_ is erroneously confirmed. As both *n*_eff_ < 30 and *H*_0_ are flagged, we now proceed to a second stage look-up table to check the result. Instead of relying on *n*_eff_ to account for red noise, we revert to using the *t*-statistic in equation (3.1), but use a look-up table generated with Monte Carlo methods in which *N* = *n* − 1, and *t*_crit_ is tabulated against both *α* and *r*_1_. The method for generating the table is described in the appendix, and values for our 5-year *h*_*i*_ window of daily samples (*n* = 1825) are provided in table 2. When we apply this test to the example in figure 3*c*, the test outcome is corrected to demonstrate that *H*_0_ is indeed false (*H*_1_ is true), which it clearly is from visual inspection.

**Table 2.** Critical *t*-values for Stage 2 of the Two-Stage Paired Thickness Test (2SPT) generated from 10 million AR(1) timeseries of length *n* = 1825 (*N* = 1824) for lag-1 autocorrelation *r*_1_ and two-sided tests at the 80% and 95% confidence intervals using the method described in the appendix. The length of the AR(1) series used here corresponds to a 5-year sequence of daily ice thickness model archives using a no-leap proleptic Gregorian calendar frequently employed in sea-ice models, but values change little by increasing the sample size to *n* = 1827 to accommodate two leap days possible within a 5-year series. Values at *r*_1_ = 0 (blue) represent the standard critical *t*-statistic for uncorrelated samples.

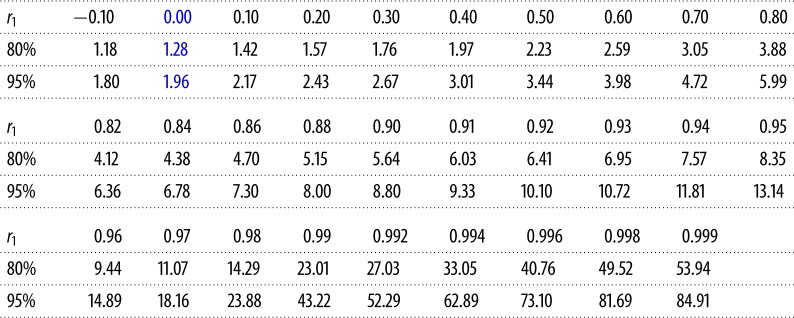

Consequently, we refer to this test as Two-Stage Paired Thickness Test (2SPT). The first stage uses the *t*-statistic in equation (3.3) to test whether or not the climate of two sea ice simulations is ostensibly the same in a model grid cell. If that test confirms that the climates are the same (*H*_0_ is true), but the effective sample size is small (*n*_eff_ < 30), we proceed to the second stage that uses the standard *t*-statistic in equation (3.1), but applies *t*_crit_ values that depend on *r*_1_. Table 2 supplies those values for our 5-year window, but we have also generated values for different series lengths shown graphically in the appendix and applied in our experiments in §4. We have generated *r*_1_-dependent *t*_crit_ values for the two-sided 80 and 95% (*α* = 0.2, 0.05) confidence intervals, but to avoid the case where integration *b* is climatically different from *a* but our test does not detect it, known as a Type II error, we use the 80% confidence interval exclusively. This makes our test extremely sensitive to code changes. Once a pass/fail result (i.e. *H*_0_/*H*_1_ confirmation) has been obtained for each model grid cell where there is sea ice, we tally the number of cells that pass as a weighted fraction of the total area of the sea-ice zone, and use that as a metric to categorize a code modification. A critical fraction, *f*_crit_, of the sea-ice zone that fails is used to divide Category II from III, and we will explore that threshold in §4.

The 2SPT test may be expressed algorithmically as follows:

*Stage* 1. For all locations on the CICE *gx1* model domain where *h*_*ai*_ or *h*_*bi*_ are greater than 0.01 m (we define this as the sea-ice zone for our purpose), determine whether *H*_0_ is true at the 80% confidence interval using equation (3.3).
*Stage* 2. If *n*_eff_ < 30 and *H*_0_ is confirmed, switch to equation (3.1) and check the result for *r*_1_ using the look-up table (table 2 and appendix), potentially correcting the results to *H*_1_ being true.
*Categorization*. Calculate the area-weighted fraction of the test regions that failed (i.e. where *H*_1_ is true). If the outcome is less than a critical fraction, *f*_crit_, the test passes as Category II, otherwise our QC algorithm stops at Category III for further review of the code.

The methods used here may be unreliable for sea-ice model variables other than thickness. Paired velocity samples may possess periodicity from inertia and tides [36,40,41], diminishing the accuracy of our underlying AR(1) approximation. Conversely, tests of paired ice concentration samples will miss changes in ice mass confined to vertical thickness evolution. For these reasons, we use neither ice concentration nor drift to test CICE code modifications.

### (c) Quadratic skill compliance test

If the new CICE code passes the test in §3b, the quality control sequence checks that spatial patterns of ice thickness from paired simulations are highly correlated and have similar variance, using a skill metric adapted from Taylor's original paper on visualizing and quantifying model performance [42]. The general skill score applicable to Taylor diagrams takes the form
3.4
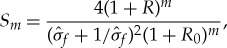
where *m* = 1 for variance-weighted skill, and *m* = 4 for correlation-weighted performance, as given in equations (4) and (5) of [42], respectively. We choose *m* = 2 to balance the importance of variance and correlation reproductions of baseline CICE simulations, and use 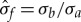 as the ratio of the standard deviations of simulations *b* and *a* sampled both spatially and temporally to test for changes to the spatial thickness pattern caused by code modifications. *R*_0_ is the maximum possible correlation between two vectors for correlation coefficient *R* calculated between thickness pairs *h*_*a*_ and *h*_*b*_ at the same place on the grid. BFB runs are perfectly correlated, *R*_0_ = 1, and the quadratic skill of run *b* relative to run *a* is
3.5
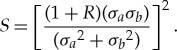
This provides a skill score between 0 and 1, and its relationship with correlation and standard deviation can be seen in the ‘Quadratic Skill’ contours shown in figure 6. The higher the score, the less difference between simulations *a* and *b*.

We apply the *S* metric to each hemisphere of a model grid by area-weighting 5 years of daily thickness samples. The hemispheric mean thickness for run *a* is
3.6
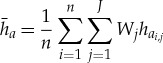
at time sample *i* and grid point *j*, and similarly for 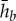. *J* is the total number of grid model points. 
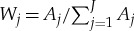
 is the weight attributed to each grid point according to its area *A*_*j*_, for all grid points within each hemisphere with one or more non-zero thicknesses in one or both sets of samples *h*_*a*_*i*,*j*__ or *h*_*b*_*i*,*j*__. The area-weighted variance for simulation *a* is
3.7
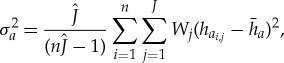
where Ĵ is the number of non-zero *W*_*j*_ weights, and *σ*_*b*_ is similar. In this context, *R* becomes a weighted correlation coefficient, calculated as
3.8
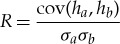
given the weighted covariance
3.9
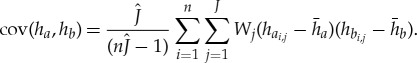


Using equations (3.5) to (3.9), the skill score *S* is calculated separately for the Northern and Southern Hemispheres. We now demonstrate, by example, that a critical value nominally set to *S*_crit_ = 0.999 is a suitable threshold separating Categories I and II from III in this quadratic skill compliance (QSC) test.

## 4. Demonstration of the quality control procedure

To demonstrate the effectiveness of the quality control procedure in categorizing code revisions, we compare twin 10-year CICE6 simulations *a* and *b* from 2000 to 2009 for scenarios where the model in *b* has been engineered to yield tiny answer changes relative to the CICE6 baseline in *a*. Each integration begins with the same initial conditions on 1 January 2000 described in §2. We present three cases, the first two corresponding to Category II scenarios (neither BFB nor climate changing), the third case providing a Category III example (non-BFB and climate changing). Each case is described here with Fortran modifications applied to CICE6 code in [5,6], where c1 and c3 are real double-precision constants equal to 1.0 and 3.0, respectively:

Ice divergence divu(i,j) used to ridge sea ice was changed in convergence and shear to divu(i,j)*(c1-c1/c3)+divu(i,j)*c1/c3 in the module ice_dyn_evp.F90.
The northeast replacement pressure variable c1ne was modified in ice_dyn_evp.F90 as c1ne=c1ne*(c1-c1/c3)+c1ne*c1/c3 immediately after its assignment.
Thermal conductivity of snow, ksno, was increased from 0.30–0.303 W m^−1^ K^−1^ in Icepack, a 1% change constituting a Category III code revision.

Modifications in RDGE and C1NE are algebraically synonymous with the CICE6 baseline, but slightly alter the numerics of the continuity and momentum equations, respectively. The KSNO case results in a Category III climate change owing to the model's strong sensitivity to the thermal conductivity of snow [43]. In fact, we define any parameter change in an existing CICE configuration as a Category III change that would first be detected during maintenance of the code repository, rather than by the 2SPT and QSC tests. Nevertheless, KSNO serves as a benchmark against which RDGE and C1NE may be compared.

**Figure 4.**
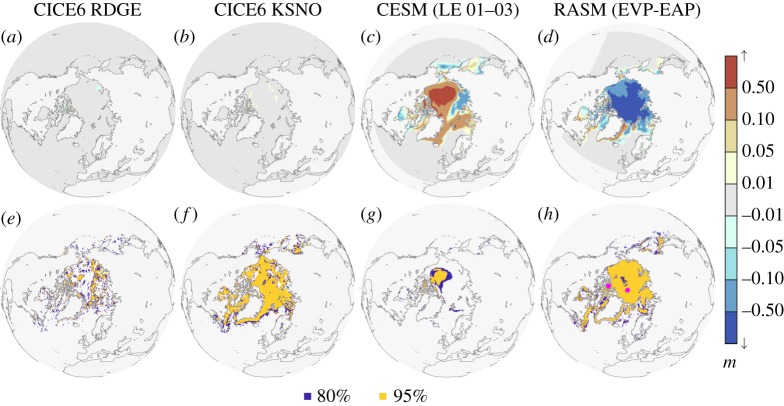
2000–2004 mean 0000 UTC daily sea-ice thickness difference 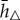 for the (*a*) RDGE and (*b*) KSNO simulations when compared with the baseline CICE6 simulation, while (*c*) compares CESM Large Ensemble members 01 and 03, and (*d*) provides the RASM EVP minus EAP comparison. (*e*–*h*) Flag regions for which the alternate hypothesis 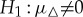 is true after Stage 2 of the 2SPT test at the 80% (blue) and 95% (amber) two-sided confidence intervals. Magenta markers in (*h*) indicate the location of the North Pole and Bathurst Island coastal time series in figure 3. Grey shading in (*a*–*d*) indicates the extent of the analysed domain for each respective model.

**Figure 5.**
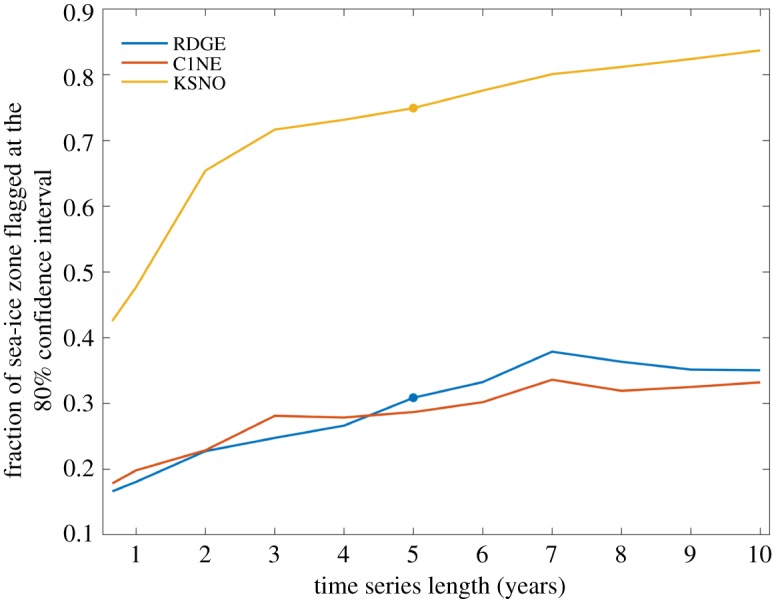
Fraction of the Northern Hemisphere CICE6 grid points with *h*_*ai*_ > 0.01 m or *h*_*bi*_ > 0.01 m for which the alternate hypothesis *H*_1_ is true at the point of Categorization in the 2SPT test and as a function of run length after model initialization on 1 January 2000. The graph is constructed from daily *h*_*ai*_ and *h*_*bi*_ values at each grid point analysed up to *n* = 240, 365, 730, 1095, 1460, 1825, 2190, 2555, 2920, 3285 and 3650 for each of the RDGE, C1NE and KSNO simulations, respectively. Blue (RDGE) and red (C1NE) traces indicate results for code changes that are neither bit-for-bit nor climate altering, whereas the yellow (KSNO) trace is the result of a tiny climate-modifying parameter change in the model. Round markers on the RDGE and KSNO traces correspond to the flagged regions mapped in figure 4*e*,*f* at *n* = 1825.

**Figure 6.**
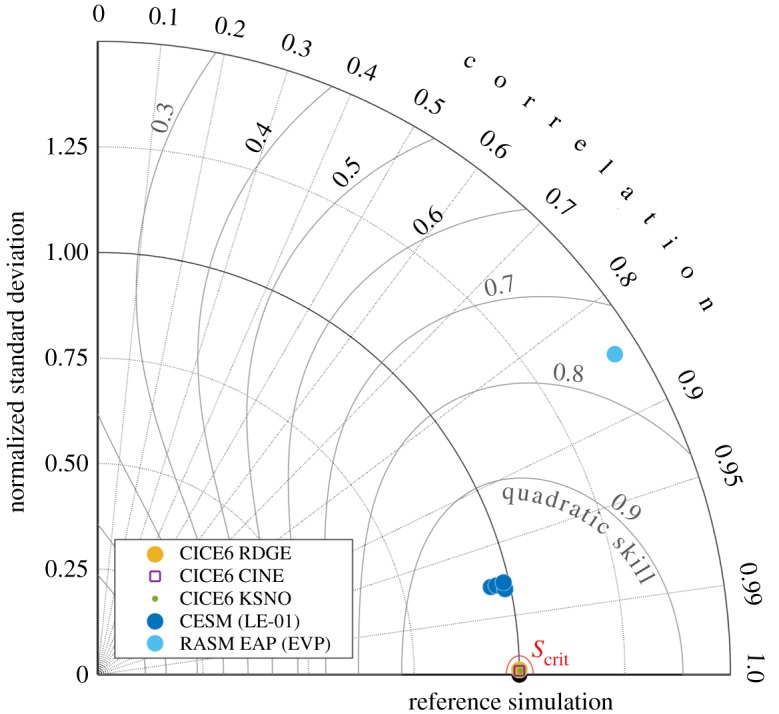
Taylor diagram illustrating the weighted quadratic skill score (*S*) for cases RDGE, C1NE and KSNO in the 2SPT Test shown in figures 4 and 5, compared with the reference simulations shown in figure 1. The critical quadratic skill score contour, *S*_crit_ = 0.999, is illustrated in red. The standard deviation is normalized against the relevant reference simulation (black) with perfect correlation. CICE6 RDGE, C1NE and KSNO markers appear in virtually the same location within the critical threshold, referenced against the CICE6 control, and the reference simulations for CESM and RASM cases are indicated in parentheses within the legend.

We seek to answer three questions using the RDGE-C1NE-KSNO suite: First, are the combined 2SPT and QSC tests sufficiently sensitive to differentiate Category I, II and III code modifications? Second, is our target 5-year test window sufficiently long for the purpose? Finally, how do the 2SPT and QSC test results from RDGE-C1NE-KSNO compare with other instances where we know that the 5-year *h*_*i*_ climate differs between paired sea-ice simulations? For this last question, we make use of the simulations from other Consortium models with clear 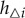 signals. Answers to each of these questions are summarized in figures 4–6, but the results of RDGE and C1NE were so similar that we omitted the latter case where appropriate (figures 4 and 6). To answer our second question, analysis of the evolution of quality control statistics is broken down into increasing time-series lengths stepped annually on the CICE no-leap calendar (*n* = 365, 730, …, 3650), and include the maximum sample count (*n* = 240) used by ZVS (see appendix). For brevity, we only present results for the northern hemisphere because if the 2SPT or QSC tests fail in one hemispheric domain, they flag a Category III review of code modification as a whole.

Figure 4*a*,*b* map the mean ice thickness differences 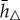 for RDGE and KSNO in our 5-year target window, with the corresponding confidence intervals flagged in figure 4*e*,*f* , respectively, after Stage 2 of the 2SPT test. In each of the RDGE and KSNO cases, less than 1.5% (8%) of the grid exceeds a mean thickness difference of 0.05 m (0.02 m), and there is no discernible trend in that statistic with increasing *n*, same for C1NE (not shown). Therefore, much of the 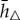 shading remains grey for RDGE and KSNO in figure 4*a*,*b*. This demonstrates why simple thickness changes cannot be used to weed out Category III cases from Category II non-BFB amendments. Instead, the fraction of the sea ice zone flagged as non-BFB emerges as a key way to differentiate Category III from II (figure 5). After 5 years, a much higher proportion (greater than 70%) of the KSNO sea-ice zone is flagged as answer changes relative to RDGE and C1NE (less than 40%). Further, figure 5 demonstrates that a 5-year window is sufficient to distinguish banal from tiny climate-changing non-BFB modifications, thus answering the first and second of our three questions. We conclude that 2SPT is sensitive to subtle code changes in each of our test cases—a combined outcome of sufficiently long series lengths (5 years) and of using a low confidence interval (80%) that helps us flag subtle 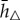 signals.

Owing to the sensitivity of the 2SPT test, we set the minimum fraction *f*_crit_ = 0.5 of the sea ice zone to be flagged as climatically different so as to acquire a category III classification. We concede that in some respects this may seem arbitrarily based on the few cases presented here. However, *f*_crit_ = 0.5 was supported by a suite of further non-BFB experiments (not shown) where we numerically but not algebraically modified CICE code, including changes to incident shortwave radiative equations. In each case, much the same results were obtained in figure 5 as in RDGE and C1NE. Our KSNO Category III benchmark undoubtedly exhibits such widespread statistical significance in the sea-ice zone because it includes changes to the column physics, rather than dynamical terms, which may be harder to detect statistically. However it is important to note that the 2SPT and QSC tests are not performed blindly nor in isolation of one another: if the BFB test fails but 2SPT passes, the nominal Category II code must pass through the final line of defence in the QSC test. Here, each of the RDGE, C1NE and KSNO simulations easily pass QSC testing by exceeding *S*_crit_ = 0.999, as shown in figure 6. The end result is that RDGE and C1NE emerge as Category II, and KSNO is assigned to Category III by 2SPT, correct in each instance.

We contrast these results against additional paired *h*_*i*_ series available from CESM and RASM that we have also used to test our methods. In this instance, the original purpose and meaning of these paired coupled simulations is irrelevant, and instead we use them to assess whether or not one simulation is ‘climate changing’ relative to another within our specific definition in §2: We wish to detect significant changes in sea-ice thickness over a substantial fraction of the pack between two 5-year *h*_*i*_ fields. Figure 4*c*,*g* compares CESM ensemble members 01 and 03 for the Arctic, and figure 6 (cobalt-blue) references ensemble members 02-05 against member 01. If, for argument's sake, the CESM ensemble members 01 and 03 were being assessed in our CICE quality control framework, they would not be flagged by the 2SPT test but instead by the QSC test as Category III because *S* < *S*_crit_ in the Taylor diagram for this model. In the same vein, we compare RASM simulations using the EVP and EAP cases to see the 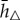 response in a second such test (figure 4*d*,*h*), with an associated skill score deflation (figure 6, light blue). In the RASM case, the change in model thickness is significant almost everywhere in the Arctic sea ice zone, and so both the 2SPT and QSC tests flag changes as Category III. The purpose here is to demonstrate that blatant differences between paired simulations can quickly be detected by developers using the the BFB-2SPT-QSC testing sequence available in CICE scripts prior to submitting code modifications for Consortium review.

## 5. Conclusion

Quality control of community sea-ice codes has, until now, been somewhat subjective, relying on a few human arbiters to judge non-BFB changes to existing model configurations. Statistical tools are now available to improve the objectivity of the process in the form of the sequence of tests described in this paper, starting with BFB certification. Existing model configurations with modified code that fail a BFB test must then not display a widespread pattern of statistically significant concentration-weighted thickness differences—the 2SPT test. Finally, the magnitude of those differences must be small, so that the modified code is hemispherically skillful relative to the version it seeks to replace—the QSC test. Importantly, the method for determining statistical significance in 2SPT requires careful consideration. Standard *t*-tests are inappropriately used in the sea-ice literature to assess model simulations, sometimes without even correcting for effective sample size. Table 2 reveals that for highly autocorrelated series, such as *h*_*i*_, critical thresholds in *t*-statistics can be more than an order of magnitude higher than is expected for *t*-tests of independent samples. In total, the BFB-2SPT-QSC testing sequence provides a computationally efficient and statistically sensitive regimen to interrogate the effect of code modifications on existing CICE configurations. It permits the assignment of clear-cut quality control categories I–III to help decide when new CICE modifications are ready to be shared with the modelling community. The methods we have presented are also broadly applicable to sea-ice model analysis, including Category IV updates requiring scientific assessment of new additions to CICE.

## Figures and Tables

**Figure 7.**
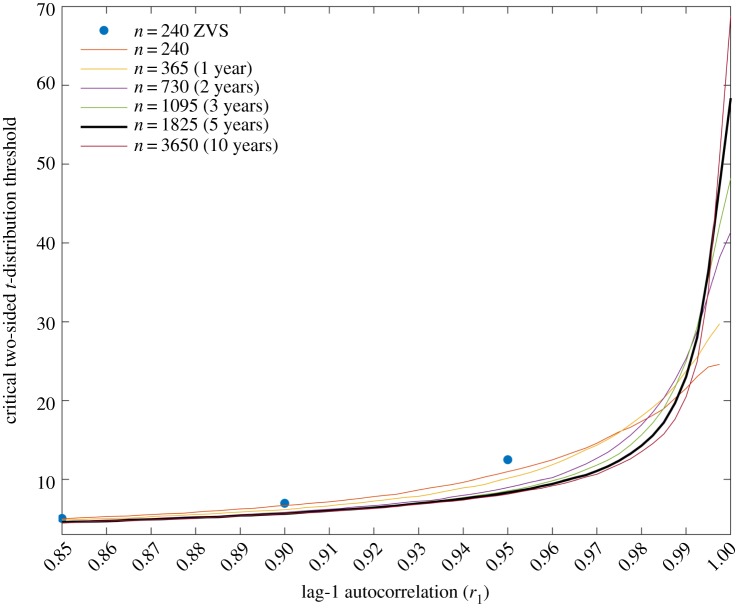
Critical *t*-statistics at the high end of the *r*_1_ scale for the 80% two-sided confidence interval generated using the method described in the appendix. Change in the statistic with increasing sample sizes is indicated for the maximum sample size explored by Zwiers and von Storch (ZVS) in [39], *n* = 240, out to the equivalent of a 10-year series of daily thickness samples from sea ice models, *n* = 3650 (no-leap calendar). Tabulated values from Zwiers & von Storch [39] appear as blue data points and are comparable with the *n* = 240 red trace generated using the large ensemble method used in this paper. The statistic for the baseline series length used by the CICE Consortium, *n* = 1825, appears in bold black.

## Data accessibility

CICE6 code used in this study is available from Hunke *et al*. [5] and its Icepack submodule can be downloaded from Hunke *et al*. [6]. CICE initialization data, documentation and code updates may be obtained at https://github.com/CICE-Consortium. Data from simulations used in this study are accessible with CICE configurations (namelist settings) for each respective model: CICE6 [44]; GOFS [45]; ECCC [46]; RASM [47,48]; CESM [23].

## Appendix A. The two-stage paired thickness look-up table

The method used to generate the look-up table for the second part of the Two-Stage Paired Thickness Test (2SPT) is similar to the technique of ZVS in [39] but differs in a few important ways. Whereas ZVS used ensemble sizes of 240 000 in their Bayesian table generation, we use 10 million AR(1) time series. While ZVS targeted low-sample counts 10≤*n*≤240 and lag-1 autocorrelations in the range −0.05≤*r*_1_≤0.95, our application caters to *r*_1_ values exceeding 0.95 with sample counts up to and exceeding 5 years of daily thickness data (*n*≥1825). Such high *r*_1_ cases exist, for example, in simulations of Canadian Archipelago ice thickness (figure 3*c*), for which autocorrelations in  can exceed 0.99. In such cases, the seven-point Monte Carlo method of ZVS did not always converge to the smooth *t*-statistic traces demonstrated in this appendix. It was found to calculate quantiles over too-broad a region of the *r*_1_ domain to capture the rapid increase in the *t*-statistic for *r*_1_ > 0.9 seen in table 2 and figure 7, and produced noisy results for low ensemble sizes of less than 1 million and high *r*_1_ values. Our experience using and testing the method in [39] led us to alter the ZVS table generation regimen. We found the method listed below more amenable to autocorrelations exceeding 0.9, and we list the revised seven-point technique here and indicate how it differs from [39]:

1. Generate an ensemble of 10 million lag-1 correlation coefficients *ρ*_1_ randomly on the interval [−0.1 1) that includes slightly negative autocorrelations. This differs from [39], for which 240 000 *ρ*_1_ samples were generated on [0 1).
2. For each randomly generated *ρ*_1_, a sample of length *n* is then generated corresponding to an AR(1) process for which *h*_*i*_ = *ρ*_1_*h*_*i*−1_ + *ε*_*i*_ for the white noise process *ε*_*i*_ and ice thickness sample *h*_*i*_ discussed in the main text.
3. *r*_1_ and *t* are then calculated from each time series ensemble member, as in [39].
4. The resulting 10 million (*r*_1_, *t*) pairs are sorted in order of increasing *r*_1_.
5. We then generate an *r*_1_*b*__ grid with values −0.1,  − 0.05, 0.0 and thereafter proceeding in steps of 0.0025 up to 1. This roughly doubles the *r*_1_*b*__ base points used by Zwiers & von Storch [39] from 200 to over 400.
6. At each of the *r*_1_*b*__ base points established in (5), we find all time series within the ensemble for which *r*_1_ is within 0.0025 of a selected *r*_1_*b*__ value, and for which there must be at least 1000 ensemble members to create a *t*-statistic corresponding to *r*_1_*b*__. This numerical approach differs from Zwiers & von Storch [39], which allowed the span of *r*_1_ values contributing to each base point to be the nearest  values of *r*_1_ from the ensemble. The square root was applied to account for curvature of the *t*-statistic seen for high *r*_1_ values in figure 7, whereas our solution uses many more ensemble members highly localized around *r*_1_*b*__ base values.
7. Compute the quantiles corresponding to the 80% and 95% two-sided confidence intervals using ensemble members satisfying the criteria in (6). This step is identical to [39].

The critical *t*-statistic generated with this revised method closely replicates the look-up table values of ZVS except where *r*_1_≥0.9, as seen for the upper *r*_1_ range in figure 7 for the 80% two-sided confidence interval. An analogous result occurs for the 95% two-sided confidence interval (not shown). The divergence from ZVS above *r*_1_ ≈ 0.9 occurs because the ensemble members contributing to each quantile are more localized about *r*_1_*b*__ points using our method than in [39]. We found the new method better suited to non-linearity in the *t*-statistic for autocorrelations exceeding *r*_1_ ≈ 0.9. Smoothly varying traces in figure 7 are evidence of the stability of our technique, from which selected values are interpolated using the two nearest *r*_1_*b*__ *t*-thresholds to create table 2. Figure 7 also demonstrates the advantage of using at least a 5-year sample size (*n*≥1825): a change in the critical *t*-value with *n* occurs more slowly for sea-ice simulations of 5 years or more than for lower sample counts. This property affirmed our decision to use a semi-decadal QC window because *r*_1_≥0.9 for up to 82% of all model grid cells analysed in the RDGE, C1NE and KSNO 2SPT tests (§4), and up to a fifth of all model grid points in those cases proceeded to Stage-2 of the test since they met the criteria *n*_eff_ < 30.

## References

[1] IPCC. 2013 Climate change 2013: the physical science basis. Contribution of working group I to the fifth assessment report of the intergovernmental panel on climate change. Cambridge, UK: Cambridge University Press (10.1017/CBO9781107415324)

[2] Metzger E *et al.* 2014 US Navy operational global ocean and Arctic ice prediction systems. Oceanography 27, 32–43. (10.5670/oceanog.2014.66)

[3] Dupont F, Higginson S, Bourdallé-Badie R, Lu Y, Roy F, Smith GC, Lemieux JF, Garric G, Davidson F 2015 A high-resolution ocean and sea-ice modelling system for the Arctic and the North Atlantic oceans. Geosci. Model Dev. 8, 1577–1594. (10.5194/gmd-8-1577-2015)

[4] Hunke EC, Lipscomb WH, Turner AK, Jeffery N, Elliott S 2015 CICE : the Los Alamos Sea Ice Model Documentation and Software User's Manual version 5.1 LA-CC-06-012. Technical Report.

[5] Hunke E *et al.* 2018 CICE-Consortium/CICE version 6.0.0.alpha (10.5281/zenodo.1205675)

[6] Hunke E *et al.* 2018 CICE-Consortium/Icepack version 1.0.2 (10.5281/zenodo.1213463)

[7] Hunke EC, Dukowicz JK 1997 An elastic-viscous-plastic model for sea ice dynamics. J. Phys. Oceanogr. 27, 1849–1867. (10.1175/1520-0485(1997)027%3C1849:AEVPMF%3E2.0.CO;2)

[8] Hunke EC 2001 Viscous–plastic sea ice dynamics with the EVP Model: linearization issues. J. Comput. Phys. 170, 18–38. (10.1006/jcph.2001.6710)

[9] Bouillon S, Fichefet T, Legat V, Madec G 2013 The elastic–viscous–plastic method revisited. Ocean Model. 71, 2–12. (10.1016/j.ocemod.2013.05.013)

[10] Tsamados M, Feltham DL, Wilchinsky AV 2013 Impact of a new anisotropic rheology on simulations of Arctic sea ice. J. Geophys. Res. Ocean 118, 91–107. (10.1029/2012JC007990)

[11] Semtner AJ Jr 1976 A model for the thermodynamic growth of sea ice in numerical investigations of climate. J. Phys. Oceanogr. 6, 379–389. (10.1175/1520-0485(1976)006%3C0379:AMFTTG%3E2.0.CO;2)

[12] Bitz CM, Lipscomb WH 1999 An energy-conserving thermodynamic model of sea ice. J. Geophys. Res. 104, 15 669–15 677. (10.1029/1999JC900100)

[13] Turner AK, Hunke EC, Bitz CM 2013 Two modes of sea-ice gravity drainage: a parameterization for large-scale modeling. J. Geophys. Res. Ocean. 118, 2279–2294. (10.1002/jgrc.20171)

[14] Holland MM, Bailey DA, Briegleb BP, Light B, Hunke EC 2012 Improved sea ice shortwave radiation physics in CCSM4: the impact of melt ponds and aerosols on Arctic Sea Ice. J. Clim. 25, 1413–1430. (10.1175/JCLI-D-11-00078.1)

[15] Hunke EC, Hebert DA, Lecomte O 2013 Level-ice melt ponds in the Los Alamos sea ice model, CICE. Ocean Model. 71, 26–42. (10.1016/j.ocemod.2012.11.008)

[16] Flocco D, Feltham DL 2007 A model of melt pond evolution on sea ice. J. Geophys. Res. C Ocean. 112, 1–14. (10.1029/2004JC002361)

[17] Holland MM, Bitz CM, Hunke EC, Lipscomb WH, Schramm JL 2006 Influence of the sea ice thickness distribution on polar climate in CCSM3. J. Clim. 19, 2398–2414. (10.1175/JCLI3751.1)

[18] Briegleb BP, Light B 2007 A delta-Eddington multiple scattering parameterization for solar radiation in the sea ice component of the community climate system model. Technical Report NCAR/TN-47, National Center for Atmospheric Research.

[19] Turner AK, Hunke EC 2015 Impacts of a mushy-layer thermodynamic approach in global sea-ice simulations using the CICE sea-ice model. J. Geophys. Res. 120, 1253–1275. (10.1002/2014JC010358)

[20] Lemieux JF, Dupont F, Blain P, Roy F, Smith GC, Flato GM 2016 Improving the simulation of landfast ice by combining tensile strength and a parameterization for grounded ridges. J. Geophys. Res. Ocean. 121, 7354–7368. (10.1002/2016JC012006)

[21] Hamman J, Nijssen B, Roberts A, Craig A, Maslowski W, Osinski R 2017 The coastal streamflow flux in the Regional Arctic System Model. J. Geophys. Res. Ocean. 122, 1683–1701. (10.1002/2016JC012323)

[22] Cassano J *et al.* 2017 Development of the Regional Arctic System Model (RASM): near-surface atmospheric climate sensitivity. J. Clim. 30, 5729–5753. (10.1175/JCLI-D-15-0775.1)

[23] Kay JE *et al.* 2015 The Community Earth System Model (CESM) Large Ensemble project: a community resource for studying climate change in the presence of internal climate variability Bull. Am. Meteorol. Soc. 96, 1333–1349. (10.1175/BAMS-D-13-00255.1)

[24] Hunke EC, Lipscomb WH 2008 CICE: The Los Alamos sea ice model. Documentation and software user's manual version 4.0. Technical Report LA-CC-06-012, Los Alamos National Laboratory.

[25] Meier W, Fetterer F, Savoie M, Mallory S, Duerr R, Stroeve J 2017 NOAA/NSIDC climate data record of passive microwave sea ice concentration, version 2. National Snow and Ice Data Center, Boulder, Colorado, USA (10.7265/N55M63M1)

[26] Hunke EC, Holland MM 2007 Global atmospheric forcing data for Arctic Ice-Ocean modeling. J. Geophys. Res. 112, C04S14 (10.1029/2006JC003640)

[27] Saha S *et al.* 2010 The NCEP climate forecast system reanalysis. Bull. Am. Meteorol. Soc. 91, 1015–1057. (10.1175/2010BAMS3001.1)

[28] Saha S *et al.* 2014 The NCEP climate forecast system version 2. J. Clim. 27, 2185–2208. (10.1175/JCLI-D-12-00823.1)

[29] Madec G, Imbard M 1996 A global ocean mesh to overcome the North Pole singularity. Clim. Dyn. 12, 381–388. (10.1007/BF00211684)

[30] Hibler WD III 1979 A dynamic thermodynamic sea ice model. J. Phys. Oceanogr. 9, 815–846. (10.1175/1520-0485(1979)009%3C0815:ADTSIM%3E2.0.CO;2)

[31] Smith GC, Roy F, Mann P, Dupont F, Brasnett B, Lemieux JF, Laroche S, Bélair S 2014 A new atmospheric dataset for forcing ice-ocean models: evaluation of reforecasts using the Canadian global deterministic prediction system. Q. J. R. Meteorol. Soc. 140, 881–894. (10.1002/qj.2194)

[32] Garric G *et al.* 2017 Performance and quality assessment of the global ocean eddy-permitting physical reanalysis GLORYS2V4. In *EGU Gen. Assem. Conf. Abstr., vol. 19 of EGU General Assembly Conference Abstracts*, p. 18776.

[33] Fetterer F, Knowles K, Meier W, Savoie M, Windnagel AK 2017 NSIDC Sea Ice Index version 3. National Snow and Ice Data Center, Boulder, Colorado, USA (10.7265/N5K072F8)

[34] Yi D, Zwally HJ 2009 Arctic Sea Ice freeboard and thickness, version 1. National Snow and Ice Data Center, Boulder, Colorado, USA (10.5067/SXJVJ3A2XIZT)

[35] Locarnini RA *et al.* 2013 World Ocean Atlas 2013, vol. 1: Temperature. Technical Report Atlas NESDIS 73, NOAA.

[36] Roberts AF, Craig A, Maslowski W, Osinski R, Duvivier A, Hughes M, Nijssen B, Cassano JJ, Brunke M 2015 Simulating transient ice-ocean Ekman transport in the Regional Arctic System Model and Community Earth System Model. Ann. Glaciol. 56, 211–228. (10.3189/2015AoG69A760)

[37] Priestley MB 1981 Spectral analysis and time series, vol. 1 and 2 Cambridge, MA: Academic Press.

[38] Wilks DS 2006 Statistical methods in the atmospheric sciences, 2nd edn Cambridge, MA: Academic Press.

[39] Zwiers FW, von Storch H 1995 Taking serial correlation into account in tests of the mean. J. Clim. 8, 336–351. (10.1175/1520-0442(1995)008%3C0336:TSCIAI%3E2.0.CO;2)

[40] Hibler W, Roberts A, Heil P, Proshutinsky A, Simmons H, Lovick J 2006 Modeling M2 tidal variability in Arctic sea-ice drift and deformation. Ann. Glaciol. 44, 418–428. (10.3189/172756406781811178)

[41] Leppäranta M, Oikkonen A, Shirasawa K, Fukamachi Y 2012 A treatise on frequency spectrum of drift ice velocity. Cold Reg. Sci. Technol. 76–77, 83–91. (10.1016/j.coldregions.2011.12.005)

[42] Taylor KE 2001 Summarizing multiple aspects of model performance. J. Geophys. Res. 106, 7183–7192. (10.1029/2000JD900719)

[43] Urrego-Blanco JR, Urban NM, Hunke EC, Turner AK, Jeffery N 2016 Uncertainty quantification and global sensitivity analysis of the Los Alamos Sea Ice Model. J. Geophys. Res. Ocean. 121, 2709–2732. (10.1002/2015JC011558)

[44] Hunke E 2018 CICE6 simulations: Quality Control for Community Based Sea Ice Model Development. Zenodo (10.5281/zenodo.1308226) PMC6107617 30126915

[45] Allard R 2018 GOFS simulations: Quality Control for Community Based Sea Ice Model Development. Zenodo (10.5281/zenodo.1308242) PMC6107617 30126915

[46] Lemieux JF 2018 ECCC simulations: Quality Control for Community Based Sea Ice Model Development. Zenodo, *Electron. Media* (10.5281/zenodo.1308965) PMC6107617 30126915

[47] Roberts A 2018 RASM simulations: Quality Control for Community Based Sea Ice Model Development. Zenodo (10.5281/zenodo.1308236) PMC6107617 30126915

[48] Roberts A 2018 2SPT Test Time Series: Quality Control for Community Based Sea Ice Model Development. Zenodo, *Electron. Media* (10.5281/zenodo.1311274) PMC6107617 30126915

